# Tissue Engineering Strategies Applied in Bone Regeneration and Bone Repair

**DOI:** 10.3390/bioengineering10060644

**Published:** 2023-05-25

**Authors:** Alexis Delpierre, Guillaume Savard, Matthieu Renaud, Gael Y. Rochefort

**Affiliations:** 1 Faculty of Dentistry, Tours University, 37000 Tours, France; alexis.delpierre@univ-tours.fr (A.D.); guillaume.savard@univ-tours.fr (G.S.); matthieu.renaud@univ-tours.fr (M.R.); 2 Department of Odontology, Tours University Hospital, 37261 Tours, France; 3 SATT Lutech TTO, Research and Valorization Department, Sorbonne University, 75012 Paris, France

Bone regeneration and repair present significant challenges in the field of regenerative medicine. Whether caused by fractures, bone diseases, or trauma, the restoration of damaged bone tissue requires innovative strategies [1]. Tissue engineering has emerged as a promising approach to address these clinical needs by developing novel strategies for bone regeneration and repair [2].

Tissue engineering strategies for bone regeneration often rely on biological approaches that aim to mimic the natural regenerative processes within the body. This includes the use of bioactive scaffolds, growth factors, and stem cells [3]. Bioactive scaffolds provide a three-dimensional structure that mimics the extracellular matrix of bone tissue, creating a favorable microenvironment for cell adhesion, proliferation, and differentiation [4]. These scaffolds can be fabricated from biocompatible materials, such as bioceramics or biodegradable polymers, which gradually degrade over time, allowing new bone tissue to form [5]. Additionally, the incorporation of growth factors, such as bone morphogenetic proteins (BMPs) or vascular endothelial growth factors (VEGFs), can stimulate bone cell recruitment, angiogenesis, and tissue regeneration [6,7]. Stem cells, including mesenchymal stem cells (MSCs) or induced pluripotent stem cells (iPSCs), offer the potential for self-renewal and differentiation into various bone cell lineages, making them valuable tools for bone tissue engineering [8].

In addition to biological approaches, mechanical strategies play a crucial role in bone regeneration and repair. Mechanical stimulation, such as through the application of biophysical forces, has been shown to enhance the healing process [9]. This can be achieved through the use of dynamic mechanical loading, which applies controlled mechanical forces to the regenerating bone tissue. These mechanical stimuli promote cell proliferation, matrix synthesis, and alignment, resulting in improved bone formation and strength [10]. Mechanical approaches can also include the design and fabrication of scaffolds with specific mechanical properties to match the requirements of the regenerated bone tissue [11]. By mimicking the mechanical environment of native bone, these scaffolds can enhance cell attachment, differentiation, and tissue integration.

The ultimate goal of tissue engineering strategies applied in bone regeneration and repair is their successful translation into clinical practice [12]. Several challenges must be addressed for effective clinical implementation, including scalability, standardization, and regulatory approval [13]. Manufacturing processes should allow the production of tissue-engineered constructs in large quantities while maintaining their quality and reproducibility. Standardized protocols and quality control measures are necessary to ensure the safety and efficacy of these therapies. Regulatory agencies play a crucial role in establishing guidelines and evaluating the safety and effectiveness of tissue-engineered products before they can be used in clinical settings [14]. Collaborations between researchers, clinicians, and regulatory bodies are essential to accelerate the translation of tissue engineering strategies into clinical applications.

As part of advanced approaches in this field, this current Special Issue of *Bioengineering* aims to explore the very latest advances in tissue engineering strategies applied to bone regeneration and repair, highlighting the potential of this field to revolutionize the treatment of bone-related conditions. This current Special Issue includes three original articles.

The first article, entitled “Mechanical Characteristics and Bioactivity of Nanocomposite Hydroxyapatite/Collagen Coated Titanium for Bone Tissue Engineering” and authored by D.J. Patty et al., explores the application of a nanocomposite coating consisting of hydroxyapatite and collagen on titanium substrates for bone tissue engineering [15]. The combination of these materials aims to enhance the physical properties and biocompatibility of titanium implants, making them suitable for bone replacement. Hydroxyapatite, a biocompatible mineral similar in composition to bone minerals, offers excellent osteoconductive properties. By mimicking the natural bone structure, hydroxyapatite coatings facilitate bone cell growth on the implant surface. The key characteristics of hydroxyapatite coatings include low porosity, strong adhesion, high crystallinity, and chemical purity. To address the cost factor associated with commercial hydroxyapatite, the article discusses the synthesis of hydroxyapatite using biogenic materials such as Pinctada maxima shells. These alternative sources offer a cost-effective solution for hydroxyapatite production. Collagen, the main protein in bones, is also incorporated into the coating to promote cell adhesion and dispersion. However, collagen’s high degradation rate and impact on mechanical properties need to be carefully considered. The study focuses on the fabrication process of hydroxyapatite/collagen coatings on titanium substrates using the electrophoresis deposition (EPD) method. EPD allows uniform coating distribution, consolidation of composites, and impregnation of porous substrates. However, the high-temperature treatment involved in the EPD process can affect collagen properties, potentially limiting its effectiveness. Mechanical characteristics of the coated titanium substrates are evaluated through compressive strength testing. The results demonstrate that the hydroxyapatite-coated titanium samples (Ti/HAp-1 and Ti/HAp-3) exhibit significantly higher compressive strength compared to uncoated titanium. However, the inclusion of collagen in the Ti/HAp/Coll coating leads to a decrease in compressive strength, suggesting a trade-off between mechanical properties and collagen’s presence. The bioactivity of the coated substrates is assessed by immersing them in simulated body fluids (SBF). The findings reveal significant apatite growth on the Ti/HAp/Coll coating, as confirmed by XRD, FTIR, and SEM-EDS analysis. This indicates that the coating has the potential to promote bone regeneration by facilitating the formation of apatite-like structures. Overall, this study highlights the potential of nanocomposite hydroxyapatite/collagen coatings on titanium substrates for bone tissue engineering applications [15]. The findings provide insights into the mechanical characteristics and bioactivity of these coatings, shedding light on their suitability and potential limitations in promoting successful bone regeneration.

In the second article, entitled “Comprehensive Studies of the Processes of the Molecular Transfer of the Momentum, Thermal Energy and Mass in the Nutrient Media of Biotechnological Industries”, the authors A.G. Novoselov et al. present a detailed examination of the molecular transport coefficients that play a crucial role in biotechnological processes [16]. They emphasize the importance of conducting complex measurements to quantitatively determine coefficients such as dynamic viscosity, thermal diffusivity, and molecular diffusion. The study utilizes rheological and thermophysical analysis techniques to investigate these coefficients under different conditions. One notable aspect of this article is the comprehensive experimental approach adopted by the authors. They employ two types of viscometers, HÖPPLER^®^ KF 3.2 (rolling-ball) and Rheotest RN 4.1 (rotary), to measure dynamic viscosity. Additionally, the thermophysical studies are conducted using the Hot Disk TPS 2500S analyzer, which allows for accurate measurements of thermal conductivity and thermal diffusivity. The measurements are performed over a wide range of temperatures and concentrations, providing valuable data for engineering calculations of hydrodynamic and heat-exchange processes in biotechnological equipment. The authors also present empirical equations for calculating the density of aqueous solutions of beet molasses. These equations contribute to a better understanding of the relationship between the concentration of dry substances and the density of the solutions at different temperatures. The diagrams illustrating the dependence of dynamic viscosity, thermal conductivity, and thermal diffusivity on shear rate, temperature, and concentration provide clear visual representations of the experimental results. Furthermore, the article highlights the significance of research for biotechnological industries, particularly in the cultivation of microorganisms for food production. The authors emphasize the importance of maintaining optimal conditions, such as temperature and nutrient supply, for the growth and reproduction of microorganisms. The findings of this study offer valuable insights into the hydrodynamic and heat-transfer processes involved in aerobic submerged cultivation. Overall, this article makes a significant contribution to the understanding of molecular transport processes in biotechnological industries. The comprehensive experimental approach, along with the empirical equations and diagrams presented, enhances our knowledge of the factors influencing momentum transfer, thermal energy transfer, and mass transfer in nutrient media [16]. The findings can be applied to improve engineering calculations and optimize biotechnological processes, ultimately leading to more efficient and sustainable food production.

The third study, entitled “Retrospective Evaluation and Framework Development of Bone Anisotropic Material Behavior Compared with Elastic, Elastic-Plastic, and Hyper-Elastic Properties” and authored by F. Hamandi et al., aimed to retrospectively evaluate bone tissue damage and develop a realistic bone model with anisotropic material properties based on CT imaging data [17]. The study considered patient demographics such as age, gender, race, BMI, height, and weight to assess their role in causing fractures. A comparison was made between the proposed bone model and previous models that used different material properties. The results showed significant differences in the anisotropic material properties of bone compared to previous unrealistic methods. Additionally, the study found variations in bone density based on gender and race, suggesting the importance of considering these factors when designing implants or suggesting therapeutic techniques. The study provides valuable insights into the evaluation of bone tissue damage and the development of an anisotropic material model for bones. By considering patient demographics, the study acknowledges the influence of factors such as age, gender, and race on bone fractures, which adds depth to the analysis. The use of CT imaging data for developing a realistic bone model is a commendable approach, as it enhances the accuracy of the predictions. A comparison with previous models using different material properties is a significant contribution to the field. By highlighting the limitations of previous methods and demonstrating the superiority of the proposed anisotropic model, the study emphasizes the importance of considering realistic material behavior in bone simulations. The finding that bone density varies based on gender and race is intriguing. However, it is essential to consider the limitations of the sample size and demographic representation in the study. The generalizability of these findings to larger populations may be limited. Additionally, the study does not delve into the underlying factors contributing to the observed variations in bone density, which could have provided further insights. Furthermore, while the study discusses the implications of the findings for implant design and therapeutic techniques, it does not provide specific recommendations or guidelines. Including practical implications would have strengthened the practical relevance of the study. Overall, this study contributes to the understanding of bone tissue behavior and emphasizes the need for realistic material modeling [17]. However, further research with larger and more diverse populations is necessary to validate the findings and explore the underlying factors affecting bone density variations. Additionally, providing practical recommendations based on the study’s findings would enhance its practical applicability.

Tissue engineering strategies applied to bone regeneration and repair show great promise in addressing the challenges associated with bone conditions. The integration of biological and mechanical approaches, along with advances in biomaterials, scaffold design, and stem cell technologies, offer exciting opportunities to develop innovative therapies that promote bone healing and regeneration. Although there are still hurdles to overcome before widespread clinical adoption, continued research and collaboration will pave the way for the successful translation of tissue engineering strategies into effective treatments for bone regeneration and repair, improving thus the lives of countless patients.

The contributions to this Special Issue take readers on a journey to topical research activities in the specific area of tissue engineering applied to bone regeneration and repair. As the guest editor for this Special Issue, I am optimistic that this specific area of research will again spark inspiration and ideas for further research and development in the field.
